# Effect of Ultrasound on the Microbial Flora and Physicochemical Parameters of Yogurt Added to Native Mexican Plants

**DOI:** 10.3390/gels11110907

**Published:** 2025-11-13

**Authors:** Luis M. Carrillo-López, Ismael Ortíz-Aguirre, América Chávez-Martínez, Luis F. Salomé-Abarca, Lorena Luna-Rodríguez, Juan M. Vargas-Romero, Ramón M. Soto-Hernández

**Affiliations:** 1 Secretaría de Ciencia, Humanidades, Tecnología e Innovación-Botánica, Colegio de Postgraduados Campus Montecillo, Carretera, Texcoco 56264, Mexico; 2 Facultad de Zootecnia y Ecología, Universidad Autónoma de Chihuahua, Chihuahua 31453, Mexico; 3 Fruticultura, Colegio de Postgraduados, Campus Montecillo, Texcoco 56264, Mexico; 4 Departamento de Biología de la Reproducción, Universidad Autónoma Metropolitana, Unidad Iztapalapa, Ciudad de México 09340, Mexico; 5 Botánica, Colegio de Postgraduados, Campus Montecillo, Texcoco 56264, Mexico

**Keywords:** high-intensity ultrasound, secondary metabolites, syneresis, water-holding capacity, color, microbiological counts, fermented milk

## Abstract

There is a growing trend in food fortification to use natural products to improve quality during production and processing. We study the effect of high-intensity ultrasound (HIU), applied at different processing times to fresh raw cow’s milk supplemented with dried plant material (DPM), on the gel fermentation kinetics and the physicochemical profile of yogurt during storage. The results showed a significant reduction in milk fermentation with the application of HIU after inoculation (INOC). The counts of *Lactobacillus delbrueckii* ssp. *bulgaricus* and *Streptococcus thermophilus* increased with the use of HIU, producing a synergistic effect in the presence of DPM due to the phenolic acids and flavonoids present. Syneresis was reduced and the water holding capacity (WHC) significantly increased in gels obtained with milk to which DPM had been added and which was sonicated after INOC. This led to the formation of a denser and more homogeneous protein network that retained more serum during storage. The luminosity of gels produced with milk sonicated at 40 °C increased, improving their appearance. However, saturation was reduced, shifting the yellow color to a neutral hue. In gels produced with non-sonicated milk, the fat separated, forming a yellow upper layer. HIU applied after INOC in milk to which DPM had been added reduced the milk processing time, producing stable and better-quality yogurts during refrigerated storage.

## 1. Introduction

Yogurt is the most popular nutrient-dense fermented dairy beverage worldwide due to its support for the human gut microbiome [1]. According to the Codex Alimentarius standard for fermented milks (CDX 243-2003), yogurt is a fermented milk characterized by the use of symbiotic cultures of *S. thermophilus* and *L. delbrueckii* subsp. *bulgaricus* [2]. Although other *Lactobacillus* species can be used as alternative cultures, the current trend is to fortify yogurts with ingredients that have beneficial effects with regard to human health [3]. Recently, the scientific and industrial community has shown increasing interest in the fortification of yogurt with natural ingredients and products (e.g., essential oils and plant extracts) due to their improved nutritional and functional properties [1]. However, compounds derived from extraction can alter the technological properties of milk; its rheological, sensory, and microbial characteristics; and the shelf life of yogurt during storage. In the study conducted by Joung et al. [4], the use of aqueous plant extracts from traditional Korean plants (*Diospyros kaki* THUNB. and *Nelumbo nucifera*) to produce “herbal” yogurts resulted in improvements in the acidification rate and a reduction in the time needed to complete fermentation, such that there was greater viability of the starter culture, higher antioxidant activity, increased water retention capacity, and increased phenolic compound content in the yogurts. Furthermore, the addition of plant extracts improved acceptability, with higher sensory scores for flavor, taste, and texture compared with controls without such extracts. Makinde et al. [5] produced yogurt using functional ingredients added before fermentation (i.e., infusions of *Cymbopogon citratus*, *Carica papaya*, *Sorghum bicolor*, and *Ocinum gratisinum*); the total polyphenol and alkaloid contents and antioxidant activity were higher in the fortified yogurts. However, pH decreased and syneresis increased during storage, thereby decreasing overall consumer acceptability. The addition of plant extracts to yogurt can have effects during the fermentation process and during cooling and storage under refrigerated conditions. In the case of milk, technological properties, including milk composition, color, stability, coagulation, and fermentation [6], can be affected by the use of plant extracts. In the study conducted by Tang et al. [7], the addition of mulberry leaves improved the acidification rate and shortened the fermentation process, as well as the total phenolic content and antioxidant capacity, but the yogurt color was light green. Thus, the addition of plant extracts to foods is multifactorial, such that there are variables related to the extract (plant material, solvent used for extraction, temperature, extraction system, etc.) and the food itself (origin, function, and degree of processing) that must be considered during the fortification process.

The extraction of bioactive compounds from plants using HIU as an assisting technology is currently being evaluated. HIU is one of the most promising methods found to date because it is considered environmentally friendly due to the high yield of the extracts, lower solvent consumption, and shorter extraction time, and is friendly to thermosensitive compounds, unlike infusions, which require longer extraction times and high temperatures [8]. Furthermore, HIU applied alone to milk before the addition of the starter culture allows obtaining fermented milks with superior sensory properties [9], improving the texture and stimulating the production of organic acids that increase volatile compounds and can improve the aroma [10]. According to Körzendörfer et al. [11], if HIU is applied during the fermentation process, softer gels are produced with less syneresis and particle formation. Conversely, in the study carried out by Abesingue et al. [12], the use of HIU was beneficial when used before inoculating milk with starter cultures, but its effect was detrimental when applied during the fermentation process in terms of aggregate formation and the reduction in gel firmness. According to Körzendörfer and Hinrichs [13], HIU modifies milk components, reduces particle size, triggers a drop in pH and water retention capacity during storage, and alters the visual appearance of yogurt (greater homogeneity). As with the extraction of bioactive compounds from plants, the effect of HIU on milk depends on the ultrasound method per se (probe or bath equipment, frequency, amplitude/intensity, time, temperature, etc.), the milk used, and the process involved.

Fortifying yogurt with plants and plant extracts is complex, such that stabilizing the properties (technological, nutritional, functional, rheological, physicochemical, textural, microbiological, and sensory) is necessary before considering the commercialization and implementation of this technology at the industrial level. In this study, a randomized block experiment was designed to evaluate the effect of two factors—the addition of the mixture of two native Mexican species, *C. ambrosioides* (commonly known as epazote) and *O. joconostle* (commonly known as xocon ostle), and the application of HIU at different processing times, before and after over-pasteurization (OP), and after INOC with starter cultures—on the behavior of *L. delbrueckii* ssp. *bulgaricus* and *S. thermophilus*, pH changes during the fermentation process, water holding capacity (WHC), syneresis, and the color of the yogurt after a 7-d storage period at 4 °C.

## 2. Results and Discussion

### 2.1. Analysis of Secondary Metabolites of DPM

**Figure 1:**
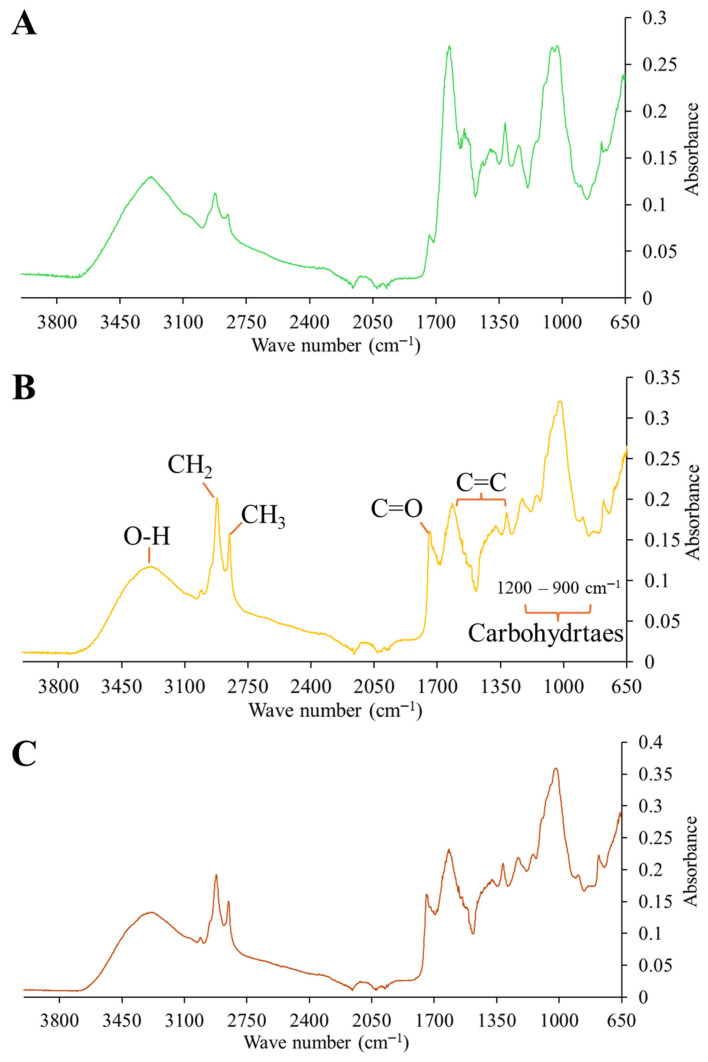
Mid-infrared spectra of (**A**) epazote (*Chenopodium ambrosioides*) leaves, (**B**) xoconostle (*Opuntia joconostle*), and (**C**) 7.25:92.50 (*w*/*w*) mixture of epazote and xoconostle leaves (40:60 ratio, respectively).

Infrared spectra showed prominent bands in the region around 3300 cm^−1^ corresponding to O–H stretching, potentially associated with carbohydrates or polyphenolic compounds [14]. Two bands were observed between 2800–2900 cm^−1^ associated with skeletal vibrations of CH_2_ (asymmetric) and CH_3_ (symmetric) groups associated with lipids or free fatty acids [15]. In the 1300–1600 cm^−1^ region, changes possibly associated with C=C vibrations within the plane of aromatic compounds were observed [16]. A band around 1700 cm^−1^, indicative of carbonyl groups (C=O) present in organic acids or free fatty acids, was observed. The 900–1200 cm^−1^ region showed numerous bands associated with CH_2_ vibrational deformations and the C–O–H and C–C–C stretching present in carbohydrates [14]. The main differences between the MIR spectra of *C. ambrosioides* and *O. joconostle* were observed in the bands at 3300, 2800–2900, and 1700–1750 and the area around 1300 cm^−1^ (Figure 1A,B). Taking into consideration the bands at 3300 and 1300–1600 cm^−1^ that correspond to hydroxyl groups and olefinic carbon stretching, this may indicate differences in polyphenolic compounds such as phenolic acids and flavonoids. Another of the most evident differences is associated with the intensities of methylene and methyl groups, which suggest differences in fatty acid content between plant materials. This would also be supported by the greater absorbance of the band at 1750 cm^−1^, corresponding to carbonyls present in acids. Due to the proportion of the plant material mixture, the infrared spectrum of the mixture is more similar to that of *O. joconostle*. Therefore, it would be expected that the content of metabolites, such as phenols and their antioxidant capacity, would also be closer to those of *O. joconostle*.

The determination of total phenols showed that *O. joconostle* has a higher total phenol content (796 mg gallic acid equivalents/g dry weight) than *C. ambrosioides* (544 mg gallic acid equivalents/g dry weight) and that the mixture of materials used as an additive in milk had an value that was intermediate (678 mg gallic acid equivalents/g dry weight) but closer to that of *O. joconostle*. Similarly, total flavonoid contents were most similar between *O. joconostle* (316 mg quercetin equivalents/g dry weight) and the mixture of plant materials (356 mg quercetin equivalents/g dry weight), whereas *C. ambrosioides* leaves showed a content about 50% lower than the other materials (179 mg quercetin equivalents/g dry weight). This was reflected in their antioxidant potentials in the DPPH assay. In this assay, *O. joconostle* extract showed the greatest free radical-scavenging capacity (57%), followed by the mixture of materials (59%), and finally *C. ambrosioides* extract as the least antioxidant (46%). Values close to ours were obtained by Abdulkader et al. [17], who reported the phenolic acid and flavonoid contents of 379.6 mg gallic acid equivalents/g dry weight and 298 mg rutin equivalents/g dry weight in hydroalcoholic extracts of *C. ambrosioides*, respectively. In the case of *O. joconostle*, Pérez-Ramírez et al. [18] reported lower content of phenolic acids and flavonoids (30–39 mg gallic acid equivalents/g fresh weight and 4–16 mg quercetin equivalents/g fresh weight, respectively) in aqueous and ethanolic extracts. These researchers used only fresh fruit peel; the flavonoid contents of 3.93 mg quercetin equivalents/g of extract have been reported in the fresh pulp [19]. Although drying influences quantification, other variables such as the processing and origin of the plant material and the specific characteristics of the drying process can influence the increase and decrease in the compounds.

### 2.2. Temperature Change (ΔT°) and pH

**Figure 2:**
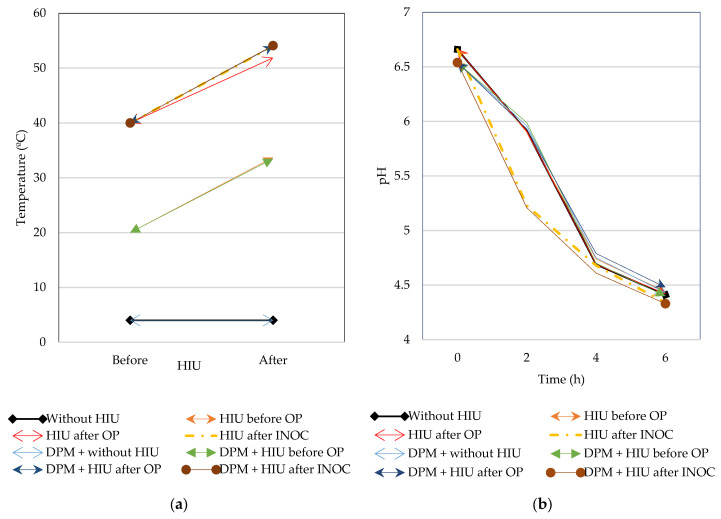
Increase in temperature (**a**) and pH evolution (**b**) during the fermentation process of milk to which DPM has been added and which has been treated with HIU; DPM—Dry-plant material; HIU—High-intensity ultrasound; OP—Over-pasteurization; INOC—Inoculation.

Treatment with HIU, regardless of the time of application, significantly increased milk temperature by up to 13–14 °C (*p* < 0.0001) (Table 1). The incorporation of DPM had no effect on the change in milk temperature (*p* = 0.1007). The combination of factors was also statistically significant (*p* < 0.0001) (Figure 2a), such that milk temperature increased by up to 15 °C in those treatments, where the initial temperature during HIU was 40 °C (after OP and after INOC). HIU increased milk temperature more at 40 °C because such milk has less dissolved air than cold milk, such that thermodynamically, less energy is needed to raise its temperature; the presence of microbubbles influences both the magnitude and location of temperature changes [20]. In warm milk, molecules have enough energy to move sufficiently fast. According to Huang et al. [21], HIU produces acoustic cavitation, a process in which microscopic bubbles form, grow, and implode, releasing a large amount of energy and causing a sudden increase in temperature. Milk temperature affects acoustic cavitation because it influences the behavior of milk components, especially fat globules and the dynamics of bubble collapse, such that preheating the milk improves the efficiency of HIU treatment by increasing the ductility and mobility of fat globules [22]. The use of thermal HIU below 57 °C can inactivate microbes, reduce fat globule size, and improve the color and stability of raw milk during storage by inactivating enzymes (alkaline phosphatase and lactoperoxidase) [23].

During the fermentation process (6 h), milk pH decreased significantly (Table 1) in all treatments (*p* < 0.0001). After the fermentation process was completed (6 h), the pH remained at approximately 4.5 after 24 h of refrigeration. During the fermentation process, lactic acid bacteria (LABs) consume milk sugar (lactose) to produce lactic acid, decreasing the pH over time and causing coagulation [24]. The blocks had a significant effect on milk pH at the beginning of the fermentation process (0 h), with milk from block 1 having a lower pH. Since it is common to find natural variation in milk batches before the application of treatments, the randomized complete block design in the present study allowed us to reduce the variability in the experimental units (components of fresh raw milk) [25]. At the beginning of the fermentation process (0 h), significant differences were found in the pH of the milk (Table 1). However, after the fermentation process, all blocks had a final pH around 4.4. Thus, treatment with HIU reduced the pH differences between milk batches, starting from the first 2 h of the fermentation process. The differences between blocks at 4 and 6 h appear to be related to the higher lactose content in blocks 1 and 2, which had a significantly lower pH value due to greater lactic acid production. Treatment with HIU during INOC, regardless of whether DPM was added or not, produced a significant decrease in milk pH after 2 h of fermentation (Figure 2b). According to Pacheco et al. [26], HIU-assisted fermentation accelerates the fermentation rate in buffalo milk to which sucrose has been added (5% *w*/*v*). This acceleration is due to the modification of the substrates. The presence of sucrose increased the availability of the fermentative substrate, favoring the metabolic activity of LAB by adapting to an environment with higher osmotic pressure [26]. In the present study, fresh raw milk to which sucrose has been added to standardize milk solids and which has been ultrasonicated during INOC favored a decrease in fermentation time of up to 34%, such that the samples reached the final pH at 4 h post-INOC. The application of HIU before or after OP did not produce differences in pH with respect to the control. Consequently, HIU may have increased LAB activity and lactic acid production and reduced pH by releasing enzymes (bacterial cell disruption and release of intracellular enzymes), which increase lactose hydrolysis and provide more substrate to LAB [27]. Compared to other non-thermal technologies, most studies with regard to yogurt fermentation involve the use of moderate pressures that cause molecular alterations (in terms of proteins and enzymes) and transiently permeabilize the membrane, improving nutrient absorption in microbial systems [28]. Unlike HIU treatment, the use of high pressures (100 MPa) can inhibit the metabolic activity of LAB and stop fermentation, but it does not reduce the fermentation time required to achieve the final pH of 4.5 [29]. In the study by Gul & Akgün [30], the fermentation time in milk intended for yogurt production was reduced by 30 min due to an increase in the starter culture population. The industrial integration of high pressures is limited by high capital costs, a lack of standardized protocols, and conformational changes in proteins that can alter texture and sensory quality [28]. Other technologies, such as pulsed electric fields, can reduce fermentation time, but by only 0.31–0.52 h [31]. Unfortunately, the use of pulsed electric fields requires a significant energy input and precise calibration. Other technologies, such as cold plasma, are focused on microbial reduction and enzyme inactivation in milk but have not been used in the lactic fermentation process [32]. Cold plasma equipment is quite complex and expensive in terms of gas consumption. In addition, this technology has a significant impact on nutrient degradation and changes in flavor and texture, as well as the generation of unwanted compounds [33].

### 2.3. Bacterial Growth Kinetics

**Figure 3:**
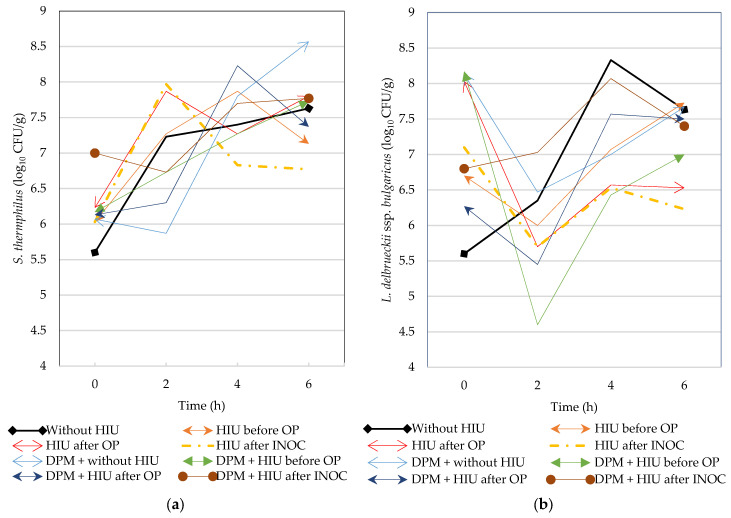
Growth of *S. thermophilus* (**a**) and *L. delbrueckii* ssp. *bulgaricus* (**b**) (log_10_ CFU/g) during the fermentation process of milk to which DPM has been added and which has been treated with HIU; DPM—Dry-plant material; HIU—High-intensity ultrasound; OP—Over-pasteurization; INOC—Inoculation; HIU—High-intensity ultrasound.

Significant differences were found in the microbial counts of *L. delbrueckii* ssp. *bulgaricus* and *S. thermophilus* at 6 h and 4–6 h, respectively, due to the blocking effect (Table 2). The addition of DPM had no effect on the counts of *L. delbrueckii* ssp. *bulgaricus* during the fermentation process (Table 2). The timing of the HIU application produced significant differences in the counts of *L. delbrueckii* ssp. *bulgaricus* at 2 h of the fermentation process. Only in the control with HIU without DPM, and in the treatment with DPM and sonication after INOC (Figure 3b), did the counts of *L. delbrueckii* ssp. *bulgaricus* increase significantly, following the pattern of normal growth kinetics. All other treatments showed significant decreases in the population of *L. delbrueckii* ssp. *bulgaricus*. These effects appear to be related to the effect of ultrasound treatment on the milk matrix. HIU increases lactose hydrolysis by increasing cell permeability and nutrient uptake through sonoporation [12], such that *L. delbrueckii* ssp. *bulgaricus* is enhanced due to the beneficial properties of DPM, such as the bioavailability of phenolics [34]. Thus, during INOC with HIU and in the presence of DPM, a synergistic effect was produced in which HIU extracted secondary metabolites from DPM that stimulated the growth of *L. delbrueckii* ssp. *bulgaricus*. Plant-derived phenols alter the metabolic processes of lactic acid fermentation depending on their concentration and the type of compound. Low concentrations of gallic acid can stimulate growth and metabolic activity by serving as a carbon source (through the hydrolysis of ester bonds and the cleavage of glycosidic bonds); however, such high concentrations alter cell wall integrity and pH, delaying carbohydrate metabolism [35,36]. Thus, polyphenols promote lactic acid bacteria (LAB) growth because they provide energy as a nutrient source; furthermore, LABs metabolize polyphenols enzymatically, producing antioxidant metabolites [36]. LABs degrade polyphenols into smaller phenol molecules to increase the total phenol content and its biological activity. According to Montijo-Prieto et al. [37], LABs promote the hydrolysis of polyphenols through the enzymatic activity of glycosidases or decarboxylase, as well as the release of phenolic compounds bound to the plant cell wall, thereby improving their bioavailability.

HIU has been shown to efficiently recover bioactive compounds from plant materials in such a way as to optimize processing in the food industry [8]. According to Carrillo-Lopez et al. [38], polar extracts of *C. ambrosioides* are rich in terpenoids and phenolic compounds (tannins and flavonoids), while *O. joconostle* can be used as a functional ingredient due to its high content in terms of fiber, sugars and organic acids, phenolic compounds and vitamin C, presenting antioxidant properties [39]. After 4 h of fermentation, the population of *L. delbrueckii* ssp. *bulgaricus* increased significantly. It is possible that the logarithmic phase starts after 2 h following INOC, and that only the lag phase is present in the control and in the DPM + HIU treatment after INOC (Figure 3b). The acidic environment may have decreased the viable bacteria count after 4 h of fermentation, such that only after 4 h of fermentation can the inoculated strains grow to the stationary phase [40].

The *S. thermophilus* population was significantly lower in block 1 after 4 h of fermentation. The composition of the milk in terms of higher fat in milk block 1 may have influenced the low fermentation rate in the first 4 h. The survival of bacterial strains is relatively higher in fat-free yogurt and lower in full-fat yogurt [41]. Milk fat is found in the form of globules in the milk, such that fragmenting these globules into smaller molecules can increase the surface area in terms of contact, and prevent fat separation in yogurt during fermentation and storage. Therefore, increasing the number of smaller globules in milk increases the number of free spaces for the *S. thermophilus* population to grow and develop; a higher fat content likely decreases the space available for increased starter bacteria counts and, consequently, for the metabolism of lactose to produce lactic acid. Thus, milk with a lower fat content (5%) can have a higher titratable acidity compared to high-fat milk (10–30%) [42]. On the other hand, bacteria respond to environmental stressors such as acidity by altering the fatty acid composition of their membrane, such that the growth rates of *S. thermophilus* can increase even in acidic environments, and a higher acid concentration can be achieved in less time [43]. Furthermore, smaller fat globules can stabilize the gel as the pH of the milk decreases [44]. Acidification leads to coagulation (precipitation and denaturation of caseins) and gel formation [45].

Furthermore, the presence of DPM significantly decreased *S. thermophilus* counts after 2 h of fermentation (Table 2); the strain adaptation to the metabolites extracted by HIU must have significantly influenced this trend. Unlike what occurred with *L. delbrueckii* ssp. *bulgaricus*, the timing of HIU application had no effect on the *S. thermophilus* population. The increases and decreases in the *S. thermophilus* population were much less abrupt than was the case with *L. delbrueckii* ssp. *bulgaricus* (Figure 3a). HIU has been documented to have a positive impact on *S. thermophilus* activity because it promotes bacterial growth [46], such that during the first 2 h an increase in *S. thermophilus* counts is observed. However, this is not the case with regard to *L. delbrueckii* ssp. *bulgaricus*. The HIU-stimulated release of β-galactosidase from *S. thermophilus* enhances lactose hydrolysis and lactic acid formation [46]. It is possible that, for this reason, the growth in the *L. delbrueckii* ssp. *bulgaricus* population occurs after 2 h of the fermentation process. *Opuntia xoconostle* has a high carbohydrate (soluble sugar) content [39]. Thus, *S. thermophilus* can metabolize lactose and sucrose and excrete galactose, while *L. delbrueckii* ssp. *bulgaricus* have variable capacities to hydrolyze sugars, such that some strains ferment galactose and sucrose, and others have less capacity to ferment these sugars [47]. In general, the decrease in milk pH during the fermentation process was directly associated with the kinetics of microbial growth. Thus, those treatments in which there was a significantly rapid reduction in pH were those in which the growth of *L. delbrueckii* ssp. *bulgaricus* and/or *S. thermophilus* was stimulated. Specifically, the HIU treatments applied after INOC were the treatments in which there was a rapid drop in pH due to the significant increase in the population of *L. delbrueckii* ssp. *bulgaricus* (with DPM) and *S. thermophilus* (without DPM). In the other treatments, the bacterial strain counts fluctuated and did so inversely, such that the final milk pH was not achieved until 6 h after the start of the fermentation process. Although HIU treatment can cause the release of β-galactosidase from the native lactic flora, the enzymatic activity can be deactivated by heat treatment (over-pasteurization). Therefore, the HIU applied before OP treatment had no effect on the pH. HIU applied after OP and INOC can accelerate the reduction in pH during yogurt fermentation due to ultrasonic cavitation, which disrupts bacterial cells and releases enzymes that hydrolyze lactose and increase acid production [24,48].

### 2.4. Physicochemical Variables

**Figure 4:**
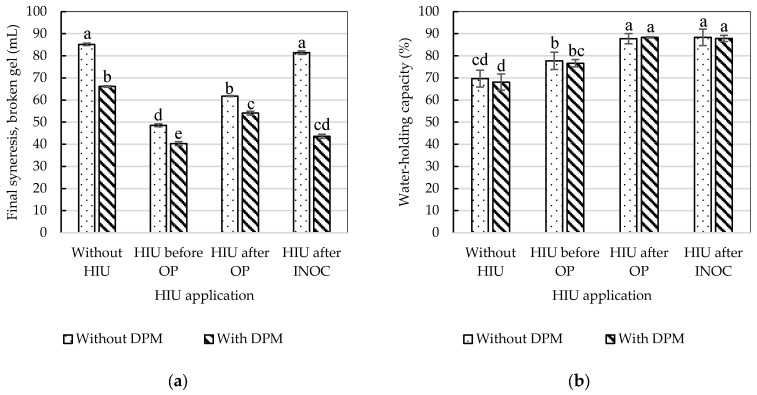
Effects of DPM × moment of the HIU application process to fresh raw milk on the final syneresis in broken gel (**a**) and WHC (**b**) of yogurt stored for 7 d at 4 °C. ^a,b,c,d,e^ Different letters in the columns within the same graph indicate statistically significant differences between treatments (Tukey’s multiple range tests assuming a significant difference at *p* < 0.05); DPM—Dry-plant material; HIU—High-intensity ultrasound; WHC—Water-holding capacity; OP—Over-pasteurization; INOC—Inoculation.

Syneresis is the most important quality parameter in yogurt because liquid expulsion during storage is undesirable. Syneresis is caused by the weakening of the gel due to shrinkage. After 24 h of storage at 4 °C, yogurts without extract and sonicated after OP and INOC showed significantly higher syneresis (Table 3, Figure 4). However, during the storage process, the gel must be stabilized, so a reduction in syneresis has been reported because HIU promotes the formation of a denser and more homogeneous protein network that retains more whey, thereby improving the WHC of yogurt [49]. This effect is due to structural changes in proteins caused by ultrasound treatment; the implosion of cavitation bubbles generated by ultrasound causes protein unfolding, exposing hydrophobic and sulfhydryl groups. Increased intermolecular hydrophobic interactions and SS bonds (through oxidation of free -SH groups) lead to the formation of protein aggregates that enhance water molecule trapping, thus improving WHC and decreasing syneresis [50]. Furthermore, polyphenols also form stable complexes with casein to create a firmer network that retains whey; the smaller fat globules generated by the ultrasound effect can more easily fit into the protein network [3].

The results showed that syneresis in block 2 was significantly lower in the broken gel after 7 d of storage at 4 °C. This effect may be due to manual agitation during gel breaking, given that the WHC did not change between blocks. The addition of DPM significantly decreased syneresis in both the broken and intact gels (Table 4). Thus, the lowest syneresis was obtained with the use of DPM in all sonication treatments (Figure 4a). The addition of fiber-rich plant materials such as purslane has been reported to reduce syneresis by up to 20% [51]. In the case of *O. joconostle*, fiber contents of up to 30–40% have been reported [39].

The application of ultrasound after OP and INOC significantly increased syneresis in the intact gel. It is possible that exposure to ultrasound at 40 °C in milk sensitized by OP treatment caused an alteration of the protein matrix, compromising the gel structure and increasing syneresis. Heating results in the denaturation of whey proteins and an association with casein molecules, such that after fermentation to pH 4.5–4.6, the casein micelles are destabilized, forming a continuous gel network [52,53]. HIU may have produced particle sizes that were too small and with a greater surface area, which increased the interaction between milk proteins and fat, but with altered whey proteins. According to Abesingue et al. [54], HIU can cause partial denaturation of whey proteins and expose hydrophilic regions to surrounding water molecules, significantly increasing syneresis. Milk sonicated before OP produced an intact gel with very low syneresis, similar to that of the control, such that HIU had no advantage in terms of reducing water release from the gel. However, in the broken gel, syneresis was significantly higher in the control and lower in the HIU treatments. Since this same behavior occurred with the addition of DPM, it is possible that the composition of the plant material contributes, together with HIU, to keeping the gel stable even after agitation, without negatively affecting the formation of the protein network. The network of casein proteins that trap whey may remain more stable, in a homogeneous mixture without lumps, without contracting after mechanical agitation. This is because whey proteins are denatured after OP and coagulate irreversibly, while caseins aggregate by isoelectric precipitation caused by the accumulation of lactic acid produced by LAB [55,56]. Other studies have also reported that fortifying yogurts with plant-based ingredients (i.e., banana and green papaya) reduces whey syneresis and improves WHC due to the formation of a strong protein network achieved by the denaturation of proteolytic enzymes by the effect of HIU, both in fresh and stored yogurts; polyphenols form soluble and stable complexes with caseins forming a firm network that retains whey and reduces syneresis [3].

**Figure 5:**
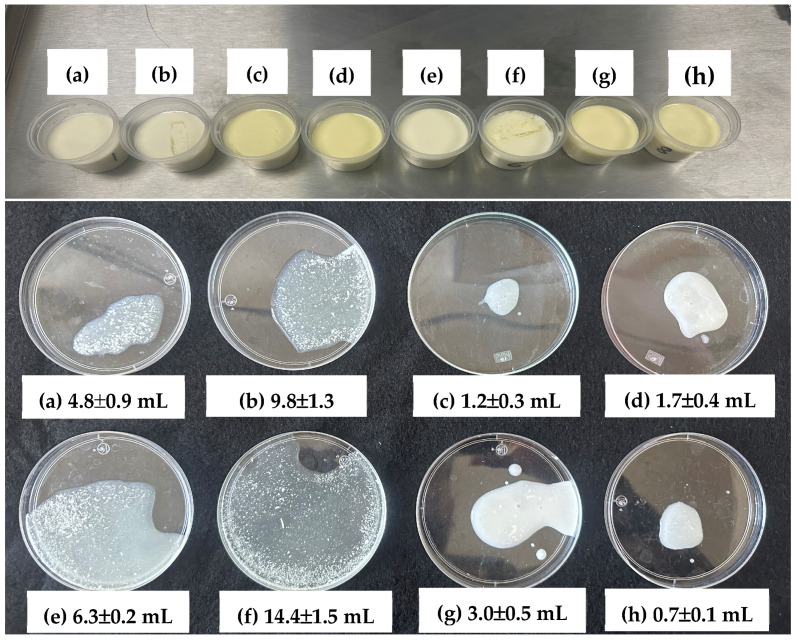
Effects of DPM × moment of the HIU application process to fresh raw milk on the color surface of yogurt stored for 7 d at 4 °C, and the initial syneresis (mL) of yogurt stored for 24 h d at 4 °C; (**a**–**d**) with DPM, (**e**–**h**) without DPM, (**a**,**e**)—HIU after OP, (**b**,**f**)—HIU after INOC, (**c**,**g**)—without HIU, (**d**,**h**)—HIU before OP. DPM—Dry-plant material; HIU—High-intensity ultrasound; OP—Over-pasteurization; INOC—Inoculation.

WHC was inversely related to syneresis in broken gels (Figure 4a,b), but not to syneresis in intact or whole gels. This appears to be due to the amount of exudated whey, which is significantly higher in broken gels and is more closely related to WHC because mechanical disruption of the protein gel structure occurs during stirring, breaking the network of dispersed particles in the whey. Vibrations during the gelation process increase the likelihood of collision and the aggregation of milk proteins, resulting in a compressed gel with syneresis. The resulting blended yogurts exhibit large particles with a compact structure, leading to reduced WHC [57]. That is, syneresis in the broken gel is more closely related to WHC than syneresis in the intact gel. WHC reflects the ability of the yogurt structure to retain water within the protein matrix. Several studies have reported that treatment involving HIU improves WHC. In the study conducted by Sıçramaz [48], WHC values of 62.3% were reported for yogurts to which pectin has been added and treated with HIU. On the other hand, Bratkič et al. [58] also reported decreases in the syneresis value and increases in WHC in yogurts fortified with *Carpobrotus edulis* extracts. Thus, in the present study, significantly greater improvements in WHC values (77–88%) were achieved when HIU was used and with the addition of DPM from *C. ambrosioides* and *O. joconostle*, due to the synergistic effect of HIU with the addition of DPM. The polyphenols present in DPM can form protein–polyphenol complexes through weak hydrophobic interactions, sometimes complemented by hydrogen bonds [56]. Thus, Zygmantaitė et al. [59] found that the addition of polyphenol-rich extract from blueberry pomace increased protein particles and accelerated acid-induced gelation time due to the formation of complexes between polyphenols and milk proteins, forming aggregated protein networks with higher viscosity and lower syneresis. Another positive effect of HIU is the reduction in fat globule size, acting as a milk homogenization method that can improve yogurt quality by increasing firmness and water holding capacity [26]. Thus, yogurts produced with milk sonicated at 40 °C (after OP and INOC) did not show fat separation (yellow coloration on the surface, Figure 5). By distributing fat evenly, HIU creates a more cohesive yogurt matrix, reducing whey separation and improving WHC [24].

**Figure 6:**
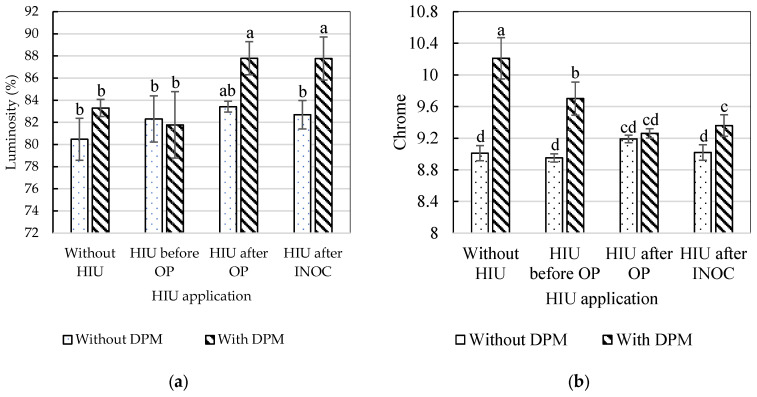
Effects of DPM × moment of the HIU application process to fresh raw milk on the luminosity (**a**) and chrome (**b**) of yogurt stored for 7 d at 4 °C. ^a,b,c,d^ Different letters in the columns within the same graph indicate statistically significant differences between treatments (Tukey’s multiple range tests assuming a significant difference at *p* < 0.05); DPM—Dry-plant material; HIU—High-intensity ultrasound; OP—Over-Pasteurization; INOC—Inoculation.

Significant changes in yogurt color parameters due to the block effect are associated with the composition of the milk batches and the color of the whey exuded during the syneresis process (Figure 5). Thus, the fresh raw milk used in each replicate (block) came from different milkings, so a significant difference between blocks was expected. The significantly higher hue angle values in block 3 indicate a more yellow hue, corresponding to the higher fat content of the fresh raw milk. However, ultrasound treatment appears to homogenize and reduce these color differences, as no significant differences in yogurt hue were found due to the timing of the HIU application. The yellow color of milk is due to the pigments it contains: carotenoids and, to a lesser extent, lutein [60]. The addition of DPM produced brighter yogurts (Table 4) and with greater color saturation (more colorful, more vivid, or intense). Regarding the timing of HIU application, yogurts from milk sonicated after OP and INOC were brighter (Table 4), and color saturation was significantly higher with HIU (regardless of the timing of application), with no significant differences in hue. Milk, then, to which DPM had been added and which had been sonicated after OP and INOC, produced the brightest yogurts (Figure 6a). The hue angle of yogurt corresponds to the yellow hue on the color wheel, with values close to 90°. The effect of HIU on color parameters depends on the application conditions. In the study by Tavşanlı et al. [61], significant improvements in the luminosity of yogurt made from milk treated with HIU (15% amplitude, 15 min) were reported; the authors attributed this effect to the increased light scattering in the visible spectrum, and the energy absorption of fat globules and casein micelles. HIU decreases fat globule sizes [62]; fat globule size reduction and increased homogenization in liquid and semi-liquid dairy products are common effects of HIU application. The effects of ultrasound on the physical structure of milk (smaller fat globule size and the release of triacylglycerides, cholesterol, and phospholipids) cause a change in light scattering and, depending on the HIU application parameters, milk color [63]. In agreement with Gregersen et al. [64], sonication powers above 30 W and temperatures above 50 °C can reduce the fat globule size from 3.39–3.89 μm to 0.37–1.9 μm. Milk carotenoids are transferred to the yogurt with minimal loss (which can occur in the whey during syneresis) and therefore contribute to the yellow coloration [65] at the end of the storage period. The use of HIU seems to reduce saturation in yogurt, shifting the yellow color to a neutral or grayish shade (less vivid or pure) compared to the addition of DPM (Figure 6b).

## 3. Conclusions

HIU and the use of plant extracts can improve fermentation kinetics and quality in yogurt. When HIU is applied after milk INOC, it is possible to reduce fermentation time and obtain the final pH of the yogurt in as little as 4 h instead of 6 h. The rapid reduction in pH is attributed to the significant increase in the counts of *L. delbrueckii* ssp. *bulgaricus* and *S. thermophilus*, which produce lactic acid from the lactose in milk. The presence of DPM produces a synergistic effect with HIU with regard to the extraction of phenolic compounds and flavonoids, which stimulate the growth of starter cultures. The physicochemical properties of yogurt can benefit from the use of milk ultrasonicated at 40 °C and to which DPM has been added; syneresis is reduced and water-holding capacity increased, with no changes in hue, but with changes in lightness and color saturation. Syneresis in the broken gel correlates better with WHC than syneresis in the intact gel because HIU promotes the formation of a denser, more homogeneous protein network, which retains more whey during storage. HIU applied after INOC and the addition of plant material reduce milk processing time and produce stable, higher-quality yogurts during refrigerated storage.

## 4. Materials and Methods

### 4.1. Preparation of Milk

This study used raw milk produced at the dairy unit of the Department of Animal Science and Ecology of the Autonomous University of Chihuahua (Chihuahua, Mexico). Milk was obtained from healthy Holstein cows reared under an intensive production system (herd of 30 cows). The milk was kept in a refrigerated storage tank (4 °C) for 3 h after milking. The milk was then analyzed (LactoScan LW, Milkotronic Ltd.^®^, Sliven, Bulgaria) in triplicate to determine the basic physicochemical parameters: titratable acidity (determined by titration with 0.1 N NaOH), pH, protein, fat, lactose, and non-fat solids (Table 5). The milk was subsequently assigned to each of the eight evaluated treatments. The study was a randomized complete block design to reduce variability within experimental units (components of fresh raw milk). Under this premise, the effects of the addition of dry plant material (DPM) were studied at two levels: with DPM and without DPM, and the timing of HIU application at four levels: without HIU, HIU before the OP process, HIU after the OP process, and HIU immediately after milk INOC, on the fermentation process and on the physicochemical properties of yogurt stored for 7 d at 4 °C (N = 3, *n* = 18).

### 4.2. Obtaining DPM

*C. ambrosioides* grows spontaneously in greenhouse chrysanthemum beds in Texcoco, Mexico. One hundred grams of mature leaves were harvested, washed three times with deionized water, and sonicated for 15 min to remove dust and soil. The material was dried in a forced-air oven at low temperature (49 °C for 96 h), ground in an agate mortar, and sieved through a 20-mesh stainless steel sieve (20 threads per inch) to homogenize particle size. The same processing was performed for *O. joconostle* using whole, recently harvested ripe fruits (including the seeds and peel of prickly pears) (48 h of storage at room temperature) purchased from a local market in Texcoco, Mexico. The mixture of *C. ambrosioides* and *O. joconostle* at a ratio of 40% and 60%, respectively, constituted the DPM in the treatments. The specific ratio of *C. ambrosioides* and *O. joconostle* was chosen based on previous studies; a higher proportion of *O. joconostle* produced protein precipitation after over-pasteurization treatment due to the high acidity of the prickly pears (pH 3.1). Both plant species are native to Mexico and are of culinary and medicinal importance.

### 4.3. Analysis of DPM

Attenuated total reflectance-coupled infrared spectroscopy (FITR-ATR) analysis was performed. Samples were analyzed directly in the solid state after being dried and pulverized (DPM). Analyses were performed on an infrared spectrometer (Cary 630, Agilent Technologies®, California, USA) equipped with an ATR accessory. For analysis, 50 mg of plant material was placed on the ATR glass. Data were acquired with 32 scans in the range of 4000–650 cm^−1^ with a nominal resolution of 4 cm^−1^. A reference measurement was taken using a clean glass before each data acquisition [66].

For the quantification of total phenols and flavonoids, 30 milligrams of plant material were extracted in 1 mL of 80% methanol by ultrasonication for 15 min. At the end of the extraction time, the samples were centrifuged at 5000 rpm for 10 min, and the supernatants were recovered. Total phenol content was quantified using the Folin–Ciocalteu technique [67]. 20 μL of extract, 1.18 mL of distilled water, 0.5 mL of Folin–Ciocalteu (1 N), and 0.3 mL of 20% Na_2_CO_3_ were mixed. The mixture was vortexed for 10 s and left to react in the dark for 1 h. Absorbance was determined at 765 nm. Quantification was performed using a gallic acid calibration curve (39–1000μg/mL). Data were expressed as mg gallic acid equivalents/gram dry weight. The correlation coefficient R^2^ for the calibration curve was 0.9991 and the slope equation was y= 0.0029x − 0.0292. A colorimetric method was used to quantify the total flavonoid content. For this purpose, 20 μL of extract was dissolved in 980 μL of 70% ethanol and mixed with 100 μL of 5% NaNO_2_. The mixture was left to react for 6 min, then 100 μL of 10% AlCl_3_·6H_2_O was added, and the mixture was homogenized by vortexing for 10 s. After 5 min of reaction, 1 mL of NaOH (0.1 M) was added, mixed, and left to react for 20 min. Absorbance was determined at 510 nm and quantified using a quercetin calibration curve (3.91ߝ250 μg/mL). Data were expressed as quercetin equivalents per gram of dry weight [68]. The correlation coefficient R^2^ for the calibration curve was 0.9996, and the slope equation was y = 0.0125x + 0.0131.

### 4.4. Design of Treatments and Yogurt Fermentation Process

The eight treatments evaluated were as follows: with DPM/without HIU, with DPM/HIU applied before OP, with DPM/HIU applied after OP, with DPM/OP applied after INOC, without DPM/without HIU, without DPM/HIU before OP, without DPM/HIU after OP, and without DPM/HIU after INOC. HIU, with or without the addition of DPM depending on the treatments evaluated, was applied using a probe system (Hielscher^®^ UP400St, Teltow, Germany) for 5 min at 100% amplitude and a frequency of 24 kHz (84 W power) with a continuous duty cycle (100% high-intensity pulse, without pause), using a 22 mm diameter probe. The ultrasound treatment was carried out at 20 °C (HIU after INOC) and 40 °C (HIU after OP), without temperature control during the process. During the ultrasonication treatment, the increase in milk temperature was monitored (Figure 2a). The HIU parameters were selected based on previous studies (sonication times greater than 10 min produce bitter flavors and rancidity in milk [69]. The ultrasonication equipment was operated according to the manufacturer’s instructions. Before applying the treatments, milk solids were adjusted by incorporating 3% partially skimmedpartially-skimmed cow’s milk powder (Carnation Clavel Nestle®, Querétaro, Mexico) and 7% refined sugar (Zucarmex^®^, Culiacán, Mexico). The treatments with DPM consisted of the addition of 0.5% (*w*/*w*) DPM. Once the solids were incorporated, the milk was subjected to a conventional over-pasteurization process at 83 ± 2 °C for 20 min. Subsequently, the milk was cooled to 43 ± 2 °C and inoculated according to the manufacturer’s instructions, using a mixed lactic culture for yogurt (*S. thermophilus* and *L. delbrueckii* subsp. *bulgaricus*, Yo-Mix 300 LYO 10 DCU (Danisco^®^, Copenhague, Denmark). The milk was incubated for 6 h at 43 ± 2 °C using an incubation chamber (Thermo Fisher Scientific®, Massachusetts, USA). The pH was monitored during the fermentation process. At the end of the incubation time, the gels were refrigerated for 24 h at 4 °C. Subsequently, the samples were divided into two groups. The first group was stored for 7 d at 4 °C for the measurement of physicochemical variables, while in the second group of samples, the gels were broken (manual shaking for 30 s) and stored for 7 d at 4 °C to determine only syneresis.

### 4.5. Microbiological Evaluations

During the milk fermentation process (0, 2, 4, and 6 h), *S. thermophilus* and *L. delbrueckii* subsp. *bulgaricus* counts were undertaken. Ten grams of each yogurt sample were aseptically homogenized in 90 mL of phosphate-buffer solution APHA, pH 7.2 NutriSelect^®^Plus (Merck KGaA, Darmstadt, Germany) and mixed with a Stomacher (Lab Blender, London, UK), operated at maximum speed for 2 min. The homogenized sample was serially diluted (1:10) in sterile phosphate-buffer solution based on Mexican Official Standards [70]. Each dilution was inoculated on specific media in triplicate. *L. delbrueckii* subsp. *bulgaricus* were plated on MRS agar and incubated anaerobically at 42 ± 2 °C for 48 h (MRS, deMan, Rogosa and Sharpe, Oxoid, Basingstoke, UK) [46]. *S. thermophilus* was plated on M17 agar (MRS, deMan, Rogosa and Sharpe, Oxoid, Basingstoke, UK) and incubated anaerobically at 37 ± 2 °C for 48 h [71]. The medium was inoculated by spreading 100 µL of diluted samples onto the agar. Colony-forming units (CFU) on the plates were counted, ranging from 10 to 200 CFU. All determinations were performed in triplicate. The number of bacteria was transformed from CFU*g^−1^ to logarithmic units (log_10_).

### 4.6. Physicochemical Properties

The pH was measured using a pH meter (Sentron, Model 1001, Woonsocket, RI, USA). Measurements were taken by inserting the electrode directly into the yogurt to a depth of 2 cm. Three readings were taken in three different areas of the sample, and the average was obtained. The pH was measured during the fermentation process (every 2 h) and after 24 days of refrigerated storage.

The WHC of the yogurts was measured in triplicate by pouring 20 g of yogurt (YW) into a cylindrical plastic cell and centrifuging for 30 min at 5000 rpm [72]. After centrifugation, the expelled whey was separated and weighed (WW). The WHC was defined as: WHC = 100 [(YW − WW)/YW]. The WHC was measured after 7 days of refrigerated storage.

Syneresis was assessed by aspirating the amount of exudated serum (mL) from the surface of the yogurt with a 10 mL pipette. Determinations were performed after 24 h of refrigerated storage (initial syneresis) and after 7 d of refrigerated storage (final syneresis) on the virgin (unbroken) gel. In another group of samples, the gel was broken after 24 h of refrigerated storage, and final syneresis was determined after 7 d of refrigerated storage.

The color space was determined using the CIE (Commission Internationale de l’Éclairage) parameters L*, a*, and b*, where L* is lightness, a*(−) is greenness, and b*(+) is yellowness. Measurements were obtained with the use of a colorimeter (Konica Minolta, CR 400, Tokyo, Japan) according to the CIE reference system. Three readings were taken from different areas of each sample of the same yogurt, and the averages were calculated. The chroma (C*) and hue angle (H*) were calculated using the expressions C* = √a*2 + b*2 and H* = tan − 1 (b*/a*), respectively. The color space was measured after 7 d of refrigerated storage.

### 4.7. Statistical Analysis

Physicochemical and microbiological data were analyzed using a completely randomized block factorial design using SAS 9.4 software (SAS Institute Inc., Cary, NC, USA), where the blocks were milking periods. Reported values are means ± standard deviation, with differences between samples being determined using Tukey’s test (significance levels *p* < 0.05). The first factor was the use of DPM (with or without DPM), while the second was the HIU application (without HIU, HIU before the OP process, HIU after the OP process, and HIU after the milk INOC), resulting in a complete factorial experiment with eight treatments and a control without extract or HIU.

## Figures and Tables

**Table 1:** Effect of blocks, addition of DPM and application of HIU on the temperature change (ΔT°) in milk and on the evolution of pH during the fermentation process; DPM—Dry-plant material; HIU—High-intensity ultrasound; OP—Over-pasteurization; INOC—Inoculation.

Tratamiento	ΔT°	pH				
Block		0 h	2 h	4 h	6 h	24 h
1	10.49 ± 6.50 ^a^	6.48 ± 0.07 ^Ac^	5.71 ± 0.28 ^Ba^	4.62 ± 0.06 ^Cb^	4.40 ± 0.06 ^Db^	4.50 ± 0.05 ^Dab^
2	10.25 ± 6.40 ^a^	6.59 ± 0.05 ^Ab^	5.78 ± 0.32 ^Ba^	4.66 ± 0.09 ^Cb^	4.38 ± 0.05 ^Db^	4.47 ± 0.07 ^Db^
3	10.30 ± 6.40 ^a^	6.74 ± 0.06 ^Aa^	5.78 ± 0.41 ^Ba^	4.80 ± 0.10 ^Ca^	4.44 ± 0.06 ^Da^	4.56 ± 0.04 ^Da^
DPM	ΔT°	0	2	4	6	24
Without	10.09 ± 6.20 ^a^	6.66 ± 0.11 ^Aa^	5.74 ± 0.32 ^Ba^	4.68 ± 0.10 ^Ca^	4.40 ± 0.05 ^Da^	4.52 ± 0.06 ^Da^
With	10.60 ± 6.40 ^a^	6.54 ± 0.12 ^Ab^	5.77 ± 0.35 ^Ba^	4.71 ± 0.12 ^Ca^	4.41 ± 0.07 ^Da^	4.50 ± 0.07 ^Da^
HIU	ΔT°	0	2	4	6	24
Not	0.0 ± 0.00 ^b^	6.60 ± 0.13 ^Aa^	5.94 ± 0.07 ^Ba^	4.72 ± 0.09 ^Ca^	4.42 ± 0.04 ^Da^	4.54 ± 0.06 ^Da^
Before OP	13.58 ± 0.56 ^a^	6.60 ± 0.13 ^Aa^	5.94 ± 0.15 ^Ba^	4.72 ± 0.11 ^Ca^	4.42 ± 0.03 ^Da^	4.55 ± 0.05 ^Da^
After OP	13.57 ± 1.79 ^a^	6.60 ± 0.13 ^Aa^	5.92 ± 0.09 ^Ba^	4.74 ± 0.13 ^Ca^	4.45 ± 0.07 ^Da^	4.51 ± 0.05 ^Dab^
After INOC	14.23 ± 0.82 ^a^	6.60 ± 0.13 ^Aa^	5.22 ± 0.09 ^Bb^	4.49 ± 0.06 ^Cb^	4.34 ± 0.03 ^Db^	4.45 ± 0.07 ^Db^

^A,B,C,D,a,b,c^ Different lowercase letters in the same column and different capital letters in the same row indicate significant statistical differences (Tukey’s multiple range tests, assuming a significant difference at *p* < 0.05).

**Table 2:** Effect of blocks, addition of DPM and application of HIU on the counts of *L. delbrueckii* ssp. *bulgaricus* and *S. thermophilus* (log_10_ CFU/g) during the milk fermentation process; DPM–Dry-plant material; HIU–High-intensity ultrasound; OP–Over-pasteurization; INOC–Inoculation.

Treatment	*L. delbrueckii* ssp. *bulgaricus* (log_10_ CFU/g)			
Block	0 h	2 h	4 h	6 h
1	7.60 ± 1.32 ^Aa^	6.00 ± 0.79 ^Ba^	7.36 ± 1.06 ^Aa^	7.61 ± 0.57 ^Aa^
2	6.79 ± 1.26 ^ABa^	5.84 ± 0.63 ^Ba^	7.10 ± 0.61 ^Aa^	6.6 ± 0.79 ^ABb^
3	6.91 ± 1.29 ^Aa^	6.04 ± 1.08 ^Aa^	7.13 ± 1.20 ^Aa^	7.43 ± 1.14 ^Aab^
DPM	0 h	2 h	4 h	6 h
Without	6.86 ± 1.21 ^ABa^	6.04 ± 0.34 ^Ba^	7.13 ± 0.98 ^Aa^	7.03 ± 0.97 ^Aa^
With	7.34 ± 1.40 ^Aa^	5.89 ± 1.14 ^Ba^	7.27 ± 0.96 ^Aa^	7.39 ± 0.89 ^Aa^
HIU	0 h	2 h	4 h	6 h
Not	6.87 ± 1.51 ^Aa^	6.41 ± 0.67 ^Aa^	7.67 ± 0.78 ^Aa^	7.65 ± 0.52 ^Aa^
Before OP	7.43 ± 1.40 ^Aa^	5.30 ± 0.77 ^Bb^	6.75 ± 1.11 ^ABa^	7.37 ± 1.04 ^Aa^
After OP	7.15 ± 1.47 ^Aa^	5.78 ± 0.36 ^Aab^	7.07 ± 0.90 ^Aa^	7.02 ± 0.89 ^Aa^
After INOC	6.95 ± 0.91 ^Aa^	6.37 ± 0.97 ^Aa^	7.30 ± 0.99 ^Aa^	6.82 ± 1.04 ^Aa^
Treatment	*S. thermophilus* (log_10_ CFU/g)			
Block	0 h	2 h	4 h	6 h
1	5.83 ± 0.66 ^Aa^	6.45 ± 1.41 ^Aa^	6.34 ± 1.28 ^Ab^	6.40 ± 1.31 ^Ab^
2	6.38 ± 0.47 ^Ba^	7.24 ± 0.89 ^Ba^	8.36 ± 0.56 ^Aa^	8.38 ± 0.65 ^Aa^
3	6.28 ± 0.46 ^Ca^	7.25 ± 0.79 ^Ba^	7.94 ± 0.34 ^Aa^	8.01 ± 0.21 ^Aa^
DPM	0 h	2 h	4 h	6 h
Without	5.98 ± 0.54 ^Ba^	7.58 ± 0.67 ^Aa^	7.34 ± 1.36 ^Aa^	7.33 ± 1.31 ^Aa^
With	6.34 ± 0.56 ^Ba^	6.38 ± 1.11 ^Bb^	7.75 ± 1.02 ^Aa^	7.86 ± 1.06 ^Aa^
HIU	0 h	2 h	4 h	6 h
Not	5.83 ± 0.36 ^Aa^	6.55 ± 0.96 ^Aa^	7.60 ± 0.59 ^Aa^	8.10 ± 0.78 ^Aa^
Before OP	6.10 ± 0.43 ^Aa^	6.93 ± 0.55 ^Aa^	7.57 ± 1.15 ^Aa^	7.43 ± 0.92 ^Aa^
After OP	6.18 ± 0.73 ^Aa^	7.08 ± 1.11 ^Aa^	7.75 ± 1.48 ^Aa^	7.58 ± 1.04 ^Aa^
After INOC	6.52 ± 0.59 ^Aa^	7.35 ± 1.61 ^Aa^	7.27 ± 1.59 ^Aa^	7.27 ± 1.89 ^Aa^

^A,B,C,a,b^ Different lowercase letters in the same column and different capital letters in the same row indicate significant statistical differences (Tukey’s multiple range tests, assuming a significant difference at *p* < 0.05).

**Table 3:** Effect of blocks, addition of DPM and application of HIU on initial syneresis (24 h), final syneresis (7 d) and WHC (7 d) of yogurt; DPM—Dry-plant material; HIU—High-intensity ultrasound; OP—Over-pasteurization; INOC—Inoculation; WHC—Water-holding capacity.

Tratamiento	Initial Syneresis (mL)	Final Syneresis		WHC
		(mL)		(%)
Block	24 h	Whole gel	Broken gel	168 h
1	5.24 ± 0.94 ^a^	8.80 ± 1.25 ^a^	62.20 ± 12.79 ^a^	80.30 ± 8.96 ^a^
2	4.83 ± 0.38 ^a^	8.50 ± 1.58 ^a^	57.20 ± 11.07 ^b^	79.40 ± 8.22 ^a^
3	5.60 ± 1.20 ^a^	9.11 ± 2.05 ^a^	61.10 ± 11.43 ^a^	82.00 ± 9.53 ^a^
Extract	0 h	2 h	4 h	6 h
Without	6.08 ± 0.46 ^a^	9.62 ± 1.84 ^a^	79.20 ± 11.79 ^a^	80.90 ± 8.58 ^a^
With	4.36 ± 0.71 ^b^	7.99 ± 1.28 ^b^	51.00 ± 6.86 ^b^	80.20 ± 8.98 ^a^
HIU	0 h	2 h	4 h	6 h
Not	2.11 ± 0.08 ^c^	3.65 ± 0.58 ^c^	75.70 ± 2.82 ^a^	68.90 ± 3.46 ^c^
Before OP	1.18 ± 0.73 ^c^	2.63 ± 0.42 ^c^	44.40 ± 1.76 ^d^	77.20 ± 2.76 ^b^
After OP	5.53 ± 0.46 ^b^	9.99 ± 0.07 ^b^	58.00 ± 2.11 ^c^	88.10 ± 1.51 ^a^
After INOC	12.07 ± 1.84 ^a^	18.95 ± 1.53 ^a^	62.5 ± 3.00 ^b^	88.10 ± 2.53 ^a^

Different lowercase letters in the same column indicate significant statistical differences (Tukey’s multiple range tests, assuming a significant difference at *p* < 0.05).

**Table 4:** Effect of blocks, addition of DPM and application of HIU on the color (CIEL*a*b*) of yogurt stored for 7 d at 4 °C (whole gel); DPM—Dry-plant material; HIU—High-intensity ultrasound; OP—Over-Pasteurization; INOC—Inoculation.

Treatment	CIE		
Block	Luminosity	Chrome	Hue Angle
1	84.76 ± 3.07 ^a^	9.43 ± 0.53 ^a^	108.9 ± 0.94 ^b^
2	82.66 ± 3.07 ^b^	9.26 ± 0.37 ^b^	109.54 ± 1.00 ^b^
3	83.64 ± 2.76 ^ab^	9.32 ± 0.38 ^ab^	111.45 ± 1.85 ^a^
DPM	0 h	2 h	4 h
Without	82.22 ± 1.75 ^b^	9.04 ± 0.11 ^b^	110.25 ± 1.84 ^a^
With	85.16 ± 3.26 ^a^	9.63 ± 0.42 ^a^	109.67 ± 1.53 ^a^
HIU	0 h	2 h	4 h
Not	81.89 ± 2.02 ^b^	9.61 ± 0.68 ^a^	110.67 ± 2.39 ^a^
Before OP	82.04 ± 2.33 ^b^	9.32 ± 0.43 ^b^	109.97 ± 2.10 ^a^
After OP	85.61 ± 2.59 ^a^	9.20 ± 0.06 ^b^	109.54 ± 0.92 ^a^
After INOC	85.22 ± 3.15 ^a^	9.19 ± 0.21 ^b^	109.68 ± 1.09 ^a^

Different lowercase letters in the same column indicate significant statistical differences (Tukey’s multiple range tests, assuming a significant difference at *p* < 0.05).

**Table 5:** Physicochemical parameters of fresh raw milk before applying the treatments.

Block	pH	Fat (%)	Protein (%)	Lactose (%)	Non-Fat-Solids (%)
1	6.48	3.69	2.93	4.4	8.09
2	6.59	3.56	2.86	4.32	7.88
3	6.74	3.62	2.74	4.29	7.73

## Data Availability

The original contributions presented in this study are included in the article. Further inquiries can be directed to the corresponding authors.

## Abbreviations

The following abbreviations are used in this manuscript:

HIU	High-intensity ultrasound
INOC	Inoculation
DPM	Dry plant material
WHC	Water-holding capacity
OP	Over-pasteurization
LAB	Lactic-acid bacteria
